# Tai Chi for osteopenic women: design and rationale of a pragmatic randomized controlled trial

**DOI:** 10.1186/1471-2474-11-40

**Published:** 2010-03-01

**Authors:** Peter M Wayne, Julie E Buring, Roger B Davis, Ellen M Connors, Paolo Bonato, Benjamin Patritti, Mary Fischer, Gloria Y Yeh, Calvin J Cohen, Danette Carroll, Douglas P Kiel

**Affiliations:** 1 Division for Research and Education in Complementary and Integrative Medical Therapies, Harvard Medical School, The Landmark Center, 401 Park Drive, Suite 22-A, Boston, MA 02215, USA; 2 Division of General Medicine and Primary Care, Department of Medicine, Beth Israel Deaconess Medical Center, 330 Brookline Ave, Boston, MA, 02215, USA; 3 Spaulding Rehabilitation Hospital, Biomotion Laboratory, 125 Nashua Street, Boston, MA 02114, USA; 4 Harvard Vanguard Medical Associates, Internal Medicine, 133 Brookline Avenue, Boston, MA 02215, USA; 5 Institute for Aging Research, Hebrew SeniorLife, 1200 Centre Street, Boston, MA 02131, USA; 6 Division of Gerontology, Beth Israel Deaconess Medical Center, 330 Brookline Ave, Boston, MA, 02215, USA

## Abstract

**Background:**

Post-menopausal osteopenic women are at increased risk for skeletal fractures. Current osteopenia treatment guidelines include exercise, however, optimal exercise regimens for attenuating bone mineral density (BMD) loss, or for addressing other fracture-related risk factors (e.g. poor balance, decreased muscle strength) are not well-defined. Tai Chi is an increasingly popular weight bearing mind-body exercise that has been reported to positively impact BMD dynamics and improve postural control, however, current evidence is inconclusive. This study will determine the effectiveness of Tai Chi in reducing rates of bone turnover in post-menopausal osteopenic women, compared with standard care, and will preliminarily explore biomechanical processes that might inform how Tai Chi impacts BMD and associated fracture risks.

**Methods/Design:**

A total of 86 post-menopausal women, aged 45-70y, T-score of the hip and/or spine -1.0 and -2.5, have been recruited from primary care clinics of a large healthcare system based in Boston. They have been randomized to a group-based 9-month Tai Chi program plus standard care or to standard care only. A unique aspect of this trial is its pragmatic design, which allows participants randomized to Tai Chi to choose from a pre-screened list of community-based Tai Chi programs. Interviewers masked to participants' treatment group assess outcomes at baseline and 3 and 9 months after randomization. Primary outcomes are serum markers of bone resorption (C-terminal cross linking telopeptide of type I collagen), bone formation (osteocalcin), and BMD of the lumbar spine and proximal femur (dual-energy X-ray absorptiometry). Secondary outcomes include health-related quality-of-life, exercise behavior, and psychological well-being. In addition, kinetic and kinematic characterization of gait, standing, and rising from a chair are assessed in subset of participants (n = 16) to explore the feasibility of modeling skeletal mechanical loads and postural control as mediators of fracture risk.

**Discussion:**

Results of this study will provide preliminary evidence regarding the value of Tai Chi as an intervention for decreasing fracture risk in osteopenic women. They will also inform the feasibility, value and potential limitations related to the use of pragmatic designs for the study of Tai Chi and related mind-body exercise. If the results are positive, this will help focus future, more in-depth, research on the most promising potential mechanisms of action identified by this study.

**Trial registration:**

This trial is registered in Clinical Trials.gov, with the ID number of NCT01039012.

## Background

Osteoporosis is a disabling condition predisposing to fractures in both women and men [1]. Nevertheless, the majority of fractures in adults occur in those with "osteopenia" (bone mineral density (BMD) only moderately lower than young normal individuals) [2]. Thus, there has been an interest in developing approaches to preventing bone loss and future fractures in this group of individuals, especially in women. Since life long drug therapy is an expensive option with uncertain consequences, non-pharmacologic therapy offers an attractive alternative. For this reason, guidelines for the treatment of osteopenia include exercise, however, there is currently no consensus regarding the optimal types and regimens of exercise for treating low BMD. Tai Chi is a mind-body exercise that is growing in popularity in the U.S. and that shows potential as an effective, sustainable, safe and practical intervention for women with low bone density. A substantial body of research already suggests Tai Chi training may reduce falls and risk factors associated with falls [3-6]. Fewer studies have evaluated the direct effects that Tai Chi may have on BMD [7]. This paper describes the rationale and design of a pilot pragmatic randomized clinical trial to gather preliminary data regarding the effectiveness of Tai Chi in reducing rates of bone turnover in post-menopausal women. It also describes an embedded substudy that aims to preliminarily explore biomechanical processes that might inform how Tai Chi impacts BMD, and ultimately other contributors to fractures related to balance and motor function.

### Osteopenia

Bone is a dynamic organ that undergoes remodeling throughout life. Bone density in women generally increases during the first three decades of life. At around 40 years of age, BMD then typically begins to decline, with more rapid changes following menopause [8], paralleling decreases in estrogen levels. However, continued bone loss in later life may also be related to other factors including decreased calcium and vitamin D intake, decreased physical activity, and age-related impairment in bone formation [9].

The 2004 Surgeon General's report on osteoporosis emphasizes that osteopenia is a serious and growing public health concern for women^1 ^The number of osteopenic women over the age of 50 in the U.S. is currently estimated at 33.6 million. Osteopenia is defined by the World Health Organization as a T-score between -1.0 and -2.5 (i.e., 1.0-2.5 standard deviations below a healthy, young white adult reference) [10]. Untreated osteopenic women are at high risk of losing additional bone and becoming osteoporotic (i.e., T score <-2.5). Osteopenic women have a 1.7 fold higher risk of fracture than women with normal bone densities [11]. While the absolute risk of fracture is higher in women with osteoporosis, the greater prevalence of osteopenia (3-fold higher than osteoporosis) results in an overall greater number of fractures in women with this level of bone fragility [11]. Low BMD-related fractures are associated with significant long-term impairment, high morbidity rates and high medical costs [12,13].

In contrast to the treatment guidelines for osteoporosis, optimal interventions for osteopenic women are not yet well-defined [14]. Current guidelines generally include the recommendation of regular exercise [1,15]. However, there is currently no consensus regarding the optimal types and regimens of exercise for treating low BMD, or for addressing other fracture-related risk factors relevant to women with osteopenia (e.g. poor balance, decreased muscle strength, diminished agility). Moreover, among post-menopausal women, compliance with conventional exercise regimens is often low, due to a variety of factors including health conditions that limit certain types of activity and lack of motivation or sustained interest [16-18]. This gap in treatment strategies for women with low bone density is emphasized in the recent Surgeon General's report on osteoporosis which stresses the need for new, creative, sustainable exercise programs that emphasize prevention [1].

### Tai Chi

Tai Chi, also known as Tai Chi Chuan and Taijiquan, has its roots in the martial arts; yet for centuries millions of Chinese have practiced its flowing, meditative movements to cultivate and maintain health. Tai Chi employs detailed regimens of physical movement, breathing techniques, and cognitive tools (both visualization and focused internal awareness) to strengthen, relax and integrate the body and mind [19-23]. Because of its reputed health benefits, apparent safety, low cost, and growing popularity, Tai Chi has become an increasingly recognized therapeutic tool by the Western medical community, and the focus of clinical and basic studies. This research is summarized in a number of recent systematic reviews [3,4,24-31].

#### Tai Chi and fracture risk

Independent of changes in BMD, Tai Chi may be of benefit to women with low bone density because of its positive effect on postural balance and fall risk. Systematic reviews [3,4,25,27] which include numerous randomized trials suggest Tai Chi practice can directly reduce risk of falls [5,6,32], and/or positively impact factors associated with postural control including fear of falling [5,33], static and/or dynamic balance [34,35], musculoskeletal strength [6,34,35] and flexibility [27,36,37], and performance of activities of daily living [38-41]. Drawing on these data, a cost-benefit analysis concluded that Tai Chi could significantly reduce costs associated with fall-related hip fractures [42]. Noteworthy across these studies is that the majority have focused on older, including frail [43] and deconditioned [44] adults, and few adverse effects have been reported. This suggests that these findings are relevant to older post-menopausal women, and that Tai Chi can be safely practiced well into later stages of life.

Research on the direct effects of Tai Chi on BMD is more limited and less conclusive [7,45]. Ten relevant studies are available in the literature, which suggest the following: (1) Long-term Tai Chi practitioners have higher BMD [46-48] than age-matched sedentary controls, and have slower rates of post-menopausal BMD decline [47]. (2) Tai Chi-naïve women who undergo Tai Chi training exhibit reduced rates of post-menopausal BMD decline. One RCT observed that dual x-ray absorptiometry (DXA) measures of BMD at the lumbar spine significantly increased (1.81%) following 10 months of Tai Chi while sedentary controls decreased (1.83%) [49]. A second RCT observed that for older women (but not men), 12 months Tai Chi resulted in maintenance of total hip BMD levels when compared to a non-exercise control that lost 2.25% of total hip BMD [50]. Also noteworthy in this study is that the beneficial effects of Tai Chi on hip BMD were equivalent to 12 months of resistance training. (3) Tai Chi is safe for peri- and post-menopausal women with no significant adverse effects reported in these studies.

While these studies suggest Tai Chi may improve BMD of post-menopausal women, results should be considered inconclusive and interpreted with caution for the following reasons: 1) The majority of the studies have design limitations. Only 5 of the 10 studies were RCTs, sample sizes were small, and information on Tai Chi interventions, qualification of instructors, recruitment and statistical methods, randomization and blinding procedures, were poorly described. 2) Studies to date have included women with a range of baseline BMD scores, from normal to severely osteoporotic. This makes its difficult to evaluate the potential benefits of Tai Chi specifically for osteopenic women. 3) Seven of the 10 studies were conducted in China and included only Asian women. It is well established that the prevalence of osteopenia and osteoporosis, and patterns of postmenopausal BMD loss vary in a predictable manner with respect to race/ethnicity--e.g. African Americans have the lowest and Caucasians and Asians the highest prevalence of osteoporosis [1].

#### Tai Chi, Mechanical Load, and Bone Mineral Density

While there is a growing understanding of the pharmacodynamic regulation of bone turnover and bone quality, much less is known about the mechanisms through which non-pharmacological interventions such as physical weight-bearing exercise and mind-body practices may impact BMD. Tai Chi is a multi-system intervention including both physical and meditative components. Understanding the mechanisms of Tai Chi's effects may inform its optimal use and provide unique insights regarding the regulation of bone dynamics and fracture risk in osteopenic women.

The reported impact of Tai Chi on retarding BMD loss has been attributed to its weight bearing characteristics. Although some systematic reviews suggest only high impact exercise favorably impact BMD in postmenopausal women with osteopenia and osteoporosis, others suggest lower impact weight bearing exercise such as walking are also effective. A Cochrane review [51] including 18 RCTs concluded aerobics, weight bearing and resistance were all equally effective in reducing bone loss in the lumbar spine, and only walking was beneficial to the hip. A recent systematic review confirmed the benefits of walking to BMD preservation at the hip but not at the spine [52]. One study that reported a long-term benefit of walking on lumbar BMD (i.e. 12 months) also reported positive impacts on markers of resorption within 3 months [53].

While Tai Chi is not a high impact exercise, it is possible that biomechanical characteristics of Tai Chi practice result in greater skeletal mechanical load than ordinary walking. Motion analysis studies of Tai Chi practitioners have reported that compared to normal gait (i.e. walking), lower extremity movements during Tai Chi have: longer cycle duration and longer duration of single-leg stance time; greater ankle, knee, and hip joint motion; a larger lateral body shift; distinct plantar pressure distributions; and greater and unique patterns of lower extremity muscle activation [54-58]. One study employing kinetic and EMG analysis reported, compared to normal gait, Tai Chi exhibits distinct peak compressive forces, larger peak shear forces in the ankle, knee and hip joints, and larger peak moments in the knee and hip joints [59]. Cross-sectional studies of elders have shown that numerous aspects of lower extremity muscle strength and endurance are comparable to joggers [60]. Other studies have reported that Tai Chi training can favorably reorganize lower extremity neuromuscular patterns, resulting in reduced excessive hip compensation and more efficient gait [61]. Together, these studies suggest that although Tai Chi is a low impact exercise, it may quantitatively and qualitatively exert skeletal mechanical loads that differ from walking and other low impact weight bearing exercises. A goal of the present pilot study is to collect preliminary biomechanical data, before and after a course of Tai Chi training, to evaluate Tai Chi's impact on lumbar and hip mechanical load, and to relate these data to clinical measures of bone turnover and changes in BMD.

### Pragmatic trials for complex interventions

Tai Chi is a complex multi-component intervention integrating neuro-musculoskeletal training, breathing, and various cognitive strategies (e.g. imagery, focused attention), often embedded within a rich psychosocial framework. Each of the physical, physiological, and psychosocial components of Tai Chi could conceivably have multiple therapeutic effects. For example, increased single leg stance time might be expected to improve lower extremity strength, impact range of motion, and enhance balance confidence. Moreover, it is likely that intervention components interact in complex ways [62]. Moreover, Tai Chi is a pluralistic intervention, represented by many different styles (e.g. Yang, Wu, Chen and Sun styles), and within each of these styles, multiple choreographed routines (e.g. 24, 48 108 form routines within the Yang style). Because of this, the clinical evaluation of Tai Chi poses unique challenges regarding choice of intervention and control groups.

It has been argued that pragmatic clinical trials are particularly informative for the study of complex interventions like Tai Chi [63]. Pragmatic trials can vary considerably in design, but their key objective is to evaluate the overall effectiveness of interventions as they are practiced in a clinical or natural setting [64,65]. Pragmatic trials generally compare the treatment of interest with an already established, credible intervention, and thus help inform choices between treatments. MacPherson [66] summarizes a number of characteristics of pragmatic trials that make them well suited for CAM therapies such as Tai Chi. These include: 1) explicit testing of the overall 'package' of components in an intervention, including the therapeutic relationship between provider and patient; 2) patients typically not blinded so their expectations are not modified; 3) intervention can be conducted in natural setting; 4) therapies not fixed and thus represent the diversity of approaches commonly employed; and 5) high external validity. Limitations of pragmatic trials include: 1) they cannot determine relative contributions of treatment components; 2) because of increased heterogeneity across interventions, larger sample sizes are generally required; and 3) reduced internal validity. Recently, Thorpe and colleagues have highlighted that the classification of pragmatic vs. more controlled explanatory study designs is not always easy and should rather be considered a continuum, as many trials include features of both design [65]. While a number of randomized trials employing pragmatic components have been conducted to evaluate the clinical effectiveness and cost-effectiveness of other complex CAM therapies such as acupuncture [67,68], surprisingly few Tai Chi trials have employed pragmatic designs. A key feature of this study is the use of a network of screened Tai Chi schools to provide Tai Chi interventions in a naturalistic setting and manner.

## Methods/Design

This trial was funded by the National Center for Complementary and Alternative Medicine (NCCAM), National Institutes of Health. The Harvard Medical School (HMS) Osher Research Center was the organizational center for the study. All clinical work was performed in Boston, MA at the Beth Israel Deaconess Medical Center and the biomechanical sub-study was conducted at the Spaulding Rehabilitation Hospital. Tai Chi was administered in the community as described below. Institutional Review Boards representing all collaborating institutions approved this study. At the time of writing this manuscript, recruitment and enrollment have been completed but interventions and outcome assessments are ongoing.

This study is a pilot randomized controlled pragmatic trial assessing the effectiveness of Tai Chi for attenuating bone loss for post-menopausal osteopenic women. Participants have been randomized to either a group-based 9-month Tai Chi program plus standard care (n = 43) or to standard care only (n = 43). Our study has been designed to address three specific aims. Aim 1 is to assess the feasibility of conducting a randomized controlled trial of Tai Chi exercise for postmenopausal osteopenic women. Feasibility is evaluated with respect to our success in recruiting subjects, administering our interventions and outcome measurements, eliciting subject compliance, and retaining subjects in the trial. Aim 2 is to collect preliminary data on the effectiveness of Tai Chi in reducing rates of BMD loss in osteopenic women. Measurements at baseline, 3 months, and 9 months include markers of bone resorption (C-terminal cross linking telopeptide of type I collagen (CTX)), bone formation (osteocalcin (OSC)), and bone density of the lumbar spine and proximal femur (dual-energy X-ray absorptiometry (DXA) at baseline and 9 months only). Aim 3 is to collect preliminary data evaluating the biomechanical, physiological, and psychological mechanisms through which Tai Chi may reduce rates of decline in BMD and fracture risks associated with osteopenia. Measurements at baseline, 3 months, and 9 months on a subset of patients (n = 16) include kinetic and kinematic characterization of gait, standing, and rising from a chair. These data will be used to model skeletal mechanical loads and postural stability. Health-related quality-of-life, exercise behavior, and psychological well-being are also being assessed over time for all participants.

**Figure 1:**
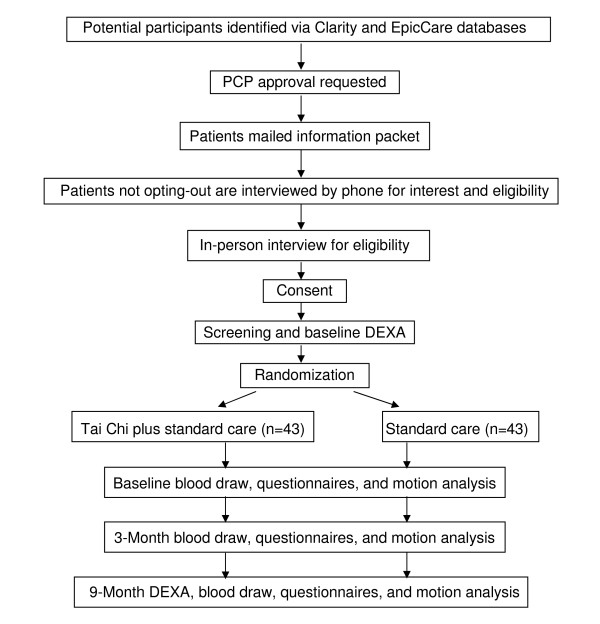
**Study flow diagram**.

Study participants randomized to the control group are offered a 3-month course of Tai Chi as a courtesy at the end of the trial. Figure 1 summarizes the overall study design and the flow of participants through the study.

### Study population and enrollment procedures

#### Study population

Participants were recruited through Harvard Vanguard Medical Associates (HVMA), a large practice of 15 clinical sites serving approximately 300,000 members in the Boston Metropolitan area. To do this we utilized the HVMA Clarity and EpicCare databases to identify all women aged 45-70 who had a DXA scan during the prior two years at a HVMA facility, with BMD of the hip (femoral neck or trochanter) and/or spine falling within the T-score range of -1.0 and -2.5. For patients who initially met these eligibility criteria based on electronic screening of HVMA records, letters were sent through the HVMA electronic medical record to primary care physicians requesting permission to contact them. If permission was granted from physicians, an introductory letter was sent to patients that describes the study and invites participation. A toll free number was provided for those interested, as well as for those who opt-out. Following a phone screen, eligible and interested participants were then scheduled for an in-person meeting conducted at the BIDMC. At the enrollment meeting, eligibility criteria were reconfirmed, and subjects provided written informed consent to participate in two stages of the study. The first stage involved a DXA screen to confirm that subjects were osteopenic (i.e. T-scores of the hip and/or spine have remained between -1.0 to -2.5). If BMD T-scores fell within the eligible range, subjects were able to continue into stage 2--the intervention stage of the trial. These subjects then completed additional baseline testing and were randomized to either the Tai Chi or the control group. During this baseline visit, randomized subjects were also asked if they wished to participate in the biomotion sub-study. This process was continued until eight participants from each study arm volunteered. Sub-study participants were then given an appointment for another baseline biomotion testing visit at Spaulding Rehabilitation Hospital, which was scheduled within two weeks of enrollment.

#### Eligibility Criteria

Participant inclusion criteria were: 1) Women ages 45-70y; BMD T-scores of the hip (fermoral neck or trochanter) and/or spine between -1.0 and -2.5; 2) post-menopausal without menses for ≥ 12 months; 3) does not exercise more than 5 days a week on average for more than 60 minutes per day.

Participant exclusion criteria were: 1) Osteoporotic (T-score < -2.5) at any site or a fracture in the past 2 years not caused by motor vehicle accident; 2) prior or current use of use of medication that increase risks of fracture (e.g. steroids, anti-convulsants, anticoagulants, lithium); 3) prior or current use of medications that modify bone metabolism (e.g. bisphosphonates, selective estrogen receptor modulators such as Raloxifene); 4) use of calcium supplements above levels suggested within the recommendations of standard care (i.e., above 1200-1500 mg); 5) current or prior year use of estrogen or calcitonin; 6) Malignancies other than skin cancer; 7) diagnosis of anorexia along with a BMI of < 17.5; 8) conditions that cause secondary osteoporosis (e.g. Cushing's syndrome, Marfan's syndrome); 9) tobacco use in past year; 10) physical or mental disabilities that will preclude informed consent or active study participation; 11) geographic or scheduling limitations that would preclude required participation in weekly Tai Chi classes and study procedures; 12) current regular practice of Tai Chi.

### Randomization procedures

Randomization assignments were generated by a computer program using a permuted block design with a variable block size. The program created a permanent record of the treatment assignment associated with each study ID. Assignments were sealed in opaque envelopes, and opened by the study staff following informed consent procedures and baseline testing.

### Interventions

#### Standard Care

Participants randomized to both the Tai Chi intervention and the control group were encouraged to follow standard of care for osteopenic women as prescribed by their primary care physicians. These guidelines vary slightly depending on T-scores as follows. For T-scores between -1.0 and -2.0, recommendations include: daily calcium (1200-1500 mg) supplements; daily vitamin D (400-800 IU); and regular exercise including weight bearing, strengthening, and balance exercise. According to HVMA guidelines, pharmacologic treatment is not indicated for this group. For women with T-scores between -2.0 and -2.5, in addition to the same supplement and exercise recommendations above, pharmacologic treatment "may be added" depending on the patient's risk profile and individual preference. Participants receiving pharmacological treatment were excluded from our trial.

#### Tai Chi intervention

Participants in the Tai Chi group receive nine months of Tai Chi training in addition to standard care. Participants were told that they were required to attend a minimum of two classes per week for the first month of the intervention, and then a minimum of one class per week for eight months thereafter (minimum class duration of one hour). They were also required to practice an additional two times per week during the first month, and three times per week thereafter (minimum of 30 minutes per session). This additional practice could take place at home, or in additional classes offered within their Tai Chi program.

Registration and enrollment in classes was facilitated by the study staff. All Tai Chi interventions have been administered at selected, reputable and long-standing Tai Chi schools throughout the Greater Boston area that meet specific guidelines described below (Table 1). A fee-for-service relationship was established between HMS and selected schools. Administering the intervention through already existing Tai Chi programs has a number of important practical and scientific advantages that increase the likelihood of success of this study. First, nine months of regular Tai Chi practice is a long and significant commitment. By allowing subjects to enroll in programs that are both located in a variety of places throughout the Greater Boston area (and thus close to homes and work places), and that offer the convenience of multiple entry-level classes per week, we hope to achieve high rates of subject recruitment, compliance, and retention. Additionally, this approach enables us to have a broad window for recruitment (i.e. rolling admission) and baseline/follow-up testing, as Tai Chi schools are eligible only if they offer ongoing enrollment or begin new entry-level courses at a minimum of every 3 months. Administering the intervention through traditional Tai Chi schools also affords a high level of ecological validity. Participants are involved in a number of traditional components of Tai Chi training that accompany the exercises themselves, including studying in traditional settings and interacting with fellow students within the school's community. These elements are often absent in fixed protocols provided in medical settings. Participation in these natural and convenient settings also make it more likely that subjects will continue studying Tai Chi once the trial is completed. This is relevant as post-study long-term adherence to Tai Chi is an outcome variable of interest in this study and will be assessed in a final audio taped exit interview. Finally, because of the inclusion of multiple Tai Chi styles and instructors, the results of this study will have good external validity--applying not only to a single protocol taught by one teacher, but to a range of approaches that share a common, well-defined set of criteria.

**Table 1:** Eligibility criteria for Tai Chi schools

*Practical Requirements*	Long standing Tai Chi school, open to the public for a minimum of 5 years
	Located within the greater Boston area
	Safe/accessible facility; phone available to dial 911 in case of medical emergency
	Offers a minimum of 2 entry-level Tai Chi classes per week throughout the year
	Entry-level classes with rolling admission, or with new classes starting at minimum every 3 months
	Senior Tai Chi instructor with a minimum of 10 years training; classes led by junior instructors supervised by a senior instructor
	Classes held in group format
	Some guidelines for home practice provided: either via verbal encouragement, handouts, or with provided media
	Verbal instruction provided in English
***Essential Curricular Requirements***	Tai Chi training based on Yang, Wu, Chen or Sun family style. All 'short' or 'long' forms acceptable if basic principles are adhered to
	Training must emphasize: 1) Relaxed continuous movement; 2) Vertical skeletal alignment; 3) Meditative intention to promote self-awareness, relaxation and concentration; 4) Some instruction in breathing techniques
	Teaching instruction incorporates modeling of Tai Chi movements and ancillary exercises, in combination with verbal and physical form correction

***Other Curricular Components (acceptable, but not required)***	Warm-up exercises conducted in the spirit of Tai Chi, such as relaxed flowing movements, flexibility, balance and self-awareness training
	Quiet sitting or standing meditation
	Gentle interactive Tai Chi exercises, for example, sensing hands
	Self massage
	Basic philosophy of Tai Chi principles as related to health

***Unacceptable Curriculum Elements (for the initial 9 month Tai Chi training period)***	Use of ancillary equipment, for example, weights or nautilus
	Training with weapons
	Sparring or wrestling
	Significant aerobic training
	Teaching of ancillary martial arts forms, for example, Kung-Fu, Ba Gua, Hsing I

***On Site Assessment***	Willing to allow Harvard Medical School study staff to observe two beginner classes in order to document adherence to eligibility criteria

### Outcome measures

#### Overview of outcome measurements

All outcome measures are assessed by study staff blind to treatment assignment. Our primary outcomes are biochemical measures of bone resorption (C-terminal cross linking telopeptide of type I collagen (CTX)), bone formation (osteocalcin (OSC)), and BMD assessed using DXA assessed at baseline and then nine months later. Secondary outcomes include measures of bone resorption and formation assessed at three months, and health-related quality of life (HRQOL), balance confidence, and exercise behavior assessed at three and nine months. Biomotion substudy participants were also evaluated to assess postural and biomechanics during activities of daily living. Table 2 provides a summary and schedule of all outcome measures.

**Table 2:** Summary and schedule of outcome variables

Outcome Variables	Sample size/group	Testing Location	Baseline	3 Months	9 Months
**Bone Density**					

DXA hip and spine	All	BIDMC	X		X

**Bone Turnover Markers**					

Serum CTX	All	BIDMC	X	X	X

Serum Osteocalcin	All	BIDMC	X	X	X

**QOL and Psychological Well-Being**					

SF 36	All	BIDMC	X	X	X

ABC Scale^¶^	All	BIDMC	X	X	X

Menopause Quality of Life Intervention	All	BIDMC	X	X	X

Expectancy Scale	All	BIDMC	X	X	X

**Exercise and Activity**					

PAR	All	BIDMC	X	X	X

Tai Chi Class Attendance	TC group	n/a	Monitored	monthly	via mail

Home Tai Chi Compliance	TC group	n/a	Monitored	monthly	via mail

Qualitative Exit Interview	TC Group	BIDMC			X

**Biomechanics**					

Gait and standing analysis	N = 16; 8/group	Spaulding Rehab	X	X	X

^¶ ^Activity Specific Balance Confidence Scale

^+ ^Physical Activity Recall Scale

#### Bone turnover markers

To study the sequence of bone remodeling activity that occurs in response to Tai Chi, we measure two bone turnover markers that reflect activity of osteoclastic resorption and osteoblastic bone formation. Type I collagen is a major component of bone, accounting for more than 90% of the organic matrix. C-terminal cross-linking telopeptide of type 1 collagen (CTX) is a breakdown product of type 1 collagen [69]. Serum levels of CTX have been shown to be sensitive and specific markers of bone resorption, and when compared with alternative resorption markers, superior [70]. Relatively short-term changes in CTX levels (e.g. 3 month) have predicted longer-term changes in BMD in pharmaceutical interventions [69-71]. We also measure serum osteocalcin (OSC), a calcium binding protein which is a major component of the non-collagenous bone matrix. During bone formation, osteoblasts synthesize OSC, which is incorporated into the extracellular bone matrix and a small fraction is released into circulation [72,73]. Serum OSC has demonstrated clinical utility as a biomarker for bone formation both in response to exercise [74] and pharmacological interventions [75]. We hypothesize that Tai Chi will have antiresorptive effects. Thus we expect to see decreases in markers of resorption and formation, since bone resorption is tightly coupled with bone formation. If Tai Chi has any anabolic potential on bone, we would expect to observe increases in both markers. By obtaining all specimens in the morning and following a 12 hour fast, diurnal variability is minimized. Processed samples of blood are stored in -80 C freezers. All specimens will thawed only once and will be analyzed as one batch at the end of the trial.

#### DXA measures of BMD

BMD of the hip and spine are measured by DXA using a QDR 4500 Discovery densitometer (Hologic, Inc., Waltham, MA) in the array (fan beam) mode. In this short trial, hip BMD is not expected to show statistically significant changes, but is being measured at baseline for eligibility determination, and at 9 months to estimate an effect size for a future study. At the screening visit, subjects undergo a single measurement of the left hip and spine. Nine-month follow-up measurements are analyzed using the "comparison" feature of the standard Hologic APEX 2.3 analysis program. The densitometry technician matches identical regions of interest (ROI) for the duplicate and follow-up examinations. Short-term in vivo estimates of the coefficients of variation for replicate baseline measurements for the total hip and lumbar spine measurements in women are expected to be about 1.5% [76]. DXA has good precision (1%) and low radiation dose (10-40 uSv) [77-79]. Longitudinal machine performance is monitored for drift using the manufacturer's phantom as is routinely done in clinical trials. The director of the DXA lab (DK) reviews all DXA analyses.

#### Health-related quality of life

As a holistic intervention, Tai Chi may simultaneously impact many components of health. Prior studies of Tai Chi have reported improvements in mood, decreased anxiety and enhancement of vigor [80-83], and some studies have reported correlations between Tai Chi-induced reduction in fall anxiety and subsequent fall probability [84].

Health-related quality-of-life (HRQOL) is a multidimensional concept that includes physical, mental, psychosocial functioning, as well as perceptions of overall health. Previous studies suggest that patients who participate in Tai Chi report significant improvements in HRQOL [38,39,44,85]. To capture health-related QOL, we are using the Medical Outcomes Survey Form (SF-36) health status survey [86]. The SF-36 has been validated for use in patients with diverse medical problems. Reported test-retest reliability coefficients range from 0.72-0.93. Fear of falling will be assessed using the Activity-specific Balance Confidence Scale (ABC Scale), which has been validated in numerous study populations [87-89]. We are also evaluating menopausal symptoms using the Menopause Quality of Life instrument [90]. One published study suggests Tai Chi can favorably manage menopausal symptoms in addition to reduced BMD [74]. Finally, a three question expectancy scale [91] to quantify participant's beliefs regarding Tai Chi is included because such beliefs have been shown to impact responses within clinical trials to other CAM modalities such as acupuncture [92].

#### Tai Chi class attendance and home practice compliance

Attendance in Tai Chi classes is recorded by having participants ask Tai Chi instructors to initial and date a wallet-size attendance card at the end of each class. Home practice is recorded using a simple practice log indicating the frequency and duration of home Tai Chi practice each week. Prepaid envelopes are provided and participants are asked to mail both class attendance cards and home practice logs at the end of each month. If attendance and/or practice logs are not submitted on time, or a participant is absent from class 3 times in a row or non-compliant with the home practice protocol (i.e. < 70% recommended), the study coordinator immediately contacts the participant, and works with them to identify and overcome any barriers related to their participation.

#### Non-Tai Chi exercise and physical activity

The goal of this study is to compare Tai Chi in addition to standard care for osteopenia--which already includes the suggestion of regular exercise--to standard care alone. Although our eligibility criteria exclude participants who state they exercise more than 5 days a week on average for more than 60 minutes per day, we actively track physical activity (other than Tai Chi) for subjects in both groups.

To measure physical activity, we employ the Seven-Day Physical Activity Recall (PAR) [93,94], recently modified to include recall of strength and flexibility activities [95]. The PAR is a general-purpose measure of physical activity widely used in clinical, epidemiological, and behavior change studies including studies of osteopenic women [96,97], and tested for validity and reliability in many populations [93,98]. The PAR estimates total energy expenditure (kcal/kg/d) using patient's recall of time spent during the previous 7 days conducting a range of activities.

#### Qualitative exit interviews

As part of the *9*-month follow-up, participants in the Tai Chi group participate in semi-structured qualitative exit interviews to further explore areas not captured in our standardized quantitative instruments. Six broad questions used in prior Tai Chi studies [39] that are being used to structure interviews include: 1) Has participation in this program had any noticeable effect on your life?; 2) Has participation in the program affected your activities of daily living in any way?; 3) Do you feel like you benefited from this program?; 4) Did you find the program enjoyable?; 5) Would you recommend Tai Chi to other women with low bone density?; 6) Do you plan to continue practice of Tai Chi? Each interview session lasts approximately 15 minutes and is audiorecorded. Tapes are then transcribed verbatim and checked against audio recordings prior to analysis. Transcribed text will be coded using the Atlas ti program (Scientific Software Development, Berlin, Germany). Interviews will be evaluated for emerging themes using a grounded theory approach [99].

### Biomotion Substudy

Specific aims of the substudy are to evaluate whether, following Tai Chi training, individuals exhibit: 1) improved balance control during quiet standing, rising from a chair, and level walking; 2) changes in joint kinetics (moment and power trajectories) during rising from a chair and level walking; and 3) improved performance on clinical tests of balance, rising from a chair, and level walking. All biomotion studies are done in the Motion Analysis Laboratory (MAL) of Spaulding Rehabilitation Hospital. All tests are performed barefoot and completed in one session of approximately 3 hours. The instrumentation in the MAL includes an eight-camera motion analysis system (VICON, Oxford, UK) and a walkway with two embedded force platforms (AMTI, Watertown, MA). For all biomechanical tests, spherical reflective markers are attached to anatomical landmarks of the feet, legs, pelvis, trunk, arms and head. The trajectories of these markers are captured by the motion analysis system and used to calculate kinematics (joint angles) and center of mass (CoM) position. The force platforms embedded in the walkway are used to collect ground reaction forces, from which we estimate center of pressure (CoP) data. CoP data are used to characterize balance control. Force platform data are used to quantify joint kinetics (moment and power trajectories) during rising from a chair and during level walking.

#### Assessment of balance control during quiet standing

We conduct tests of quiet standing balance during which subjects are asked to stand on a force platform for 40 s with arms by their side, feet shoulder-width apart and their eyes closed. The CoP trajectory is captured during each of 10 trials. After 5 trials, the subject is allowed to take a short break and sit down.

A scatter plot of the anteroposterior (AP) and mediolateral (ML) displacement of the CoP, called stabilogram, is analyzed for each trial. Balance control during the quiet standing trials is characterized via both traditional and random-walk sway parameters [100,101].

We compute the following traditional sway parameters, which describe geometric features of the stabilogram: the mean stabilogram radius (measured in millimeters - mm), the area swept by the stabilogram (mm^2^), the maximum radius of sway (mm), and the range of the AP and ML excursions (mm). We hypothesize that we will observe a reduction in postural sway, as reflected by a decrease in traditional sway parameter values thus indicating a tighter control of balance following Tai Chi training [6,34].

We also estimate three random-walk sway parameters: the critical mean square displacement ⟨Δr^2^⟩_c _(measured in square millimeters - mm^2^), the effective long-term diffusion coefficient D_rl _(mm^2^/s), and the long-term scaling exponential H_rl_. The conceptual framework underlying this analysis is based on the assumption that, during quiet standing, the postural control system uses open-loop and closed-loop control schemes over short-term and long-term intervals respectively [102]. Over short-term intervals, the body tends to drift away from a relative equilibrium point. Over long-term intervals, the body tends to return to a relative equilibrium point. The critical mean square displacement characterizes the threshold at which the postural control system switches from one control strategy to the other. The effective long-term diffusion coefficient and the long-term scaling exponential characterize the behavior of the postural control system in the open-loop and closed-loop control conditions respectively. We hypothesize that a decrease in all three random-walk sway parameters will be observed following Tai Chi training indicating a more tightly regulated system [101].

#### Changes in joint kinetics and dynamic balance control during chair rise

We test participants' ability to rise from sitting on a chair to a standing position. Subjects sit on a chair with their feet positioned about shoulder-width apart on a force platform. Subjects are asked to rise to a standing position and remain standing for about 5 s. They are allowed to rest their hands on their thighs while standing but are not allowed to use their arms to push off from the chair. Subjects are asked to perform this task 10 times and are given a break after 5 trials. The kinematics of upper and lower body movement is characterized by joint angles. The kinetics is described by joint moment and power trajectories. We assess the dynamic control of balance of each subject during the chair rise using parameters describing the relative trajectories of the CoM and CoP. We hypothesize that we will observe changes in joint kinetics and a more tightly control displacement between CoM and CoP after Tai Chi training.

#### Changes in joint kinetics and dynamic balance control during gait

The comfortable walking speed of each subject while walking barefoot is first determined during the baseline assessment. Subjects are asked to walk at this speed (± 10%) during the gait assessments at the baseline, 3-month and 9-month follow-up sessions. This will allow longitudinal within-subject comparisons of changes in gait kinematics and kinetics for the duration of each subject's participation in the trial. We collect up to 20 walking trials with good foot contacts on the force platforms. During each gait cycle, joint kinematics and kinetics will be characterized by deriving joint angle parameters and joint moment and power trajectories respectively. We will also assess dynamic stability during the walking trials by analyzing the path of the body CoM. We expect Tai Chi-induced adaptations in joint kinematics and kinetics to be associated with improved BMD [103,104]. Furthermore, we anticipate that a more tightly controlled CoM trajectory [105] will be observed following Tai Chi training [61].

#### Clinical tests

Subjects are asked to perform 5 clinical tests. The timed tandem walk [106], the Timed Up and Go test (TUG) [107,108], the Berg Balance Scale (BBS) [109], the timed one-legged stance test [110,111], and the timed chair rise test [112]. These tests have been shown to discriminate balance ability, to be predictive of falls, and to be related to muscle strength [109-113]. They represent a good range of subjective and objective clinical tests with a variety of balance-related tasks - walking, turning, and reaching - and both timed (continuous) and scored (discrete) measures. Subjects are given rest breaks between each test and trials of a test. We hypothesize that improvements in all the clinical test results will be observed following Tai Chi training [113].

### Adverse Events

Adverse events are monitored through reports from the school instructors, reports from participants, and through systematic monthly safety calls to participants conducted by study staff. Each school was presented a safety and adverse event policy along with forms to report events that occurred in class or that were otherwise reported by participants. All study participants were informed of the study's safety policy and given adverse event forms at baseline. Study staff called participants in both the Tai Chi and control group twice during the first month and monthly for the remaining 8 months to ask if they had any questions or had any safety issues related to their participation in the study.

### Statistical Analysis, Sample Size and Power

#### Statistical analysis

Specific Aim 1: To assess the feasibility of conducting a trial of Tai Chi in osteopenic women, we are using screening logs to determine the proportion of screened women who are eligible and the proportion of eligible women who enroll in the study. We track factors that make subjects ineligible, and for unwilling eligible subjects, reasons for not participating. We measure compliance through attendance at Tai Chi classes and self-reported home exercise logs. Participants who attend at least 80% of target number of classes and comply with 70% of prescribed home Tai Chi practice are considered compliant. Levels of exercise compliance will also be used as covariates in post-hoc analyses of all outcome variables (see below). We also track the proportion of participants in each group who complete each of the follow-up visits. Overall, we will consider further study feasible if at least 75% of intervention participants are compliant and at least 80% of participants in each group complete all study evaluations (i.e., baseline, 3-month and 9-month).

Specific Aim 2. The primary clinical outcomes are changes from baseline in CTX and osteocalcin (3 and 9 months), and BMD (9 months) assessed with DXA. Each outcome will be measured as the percentage change from the baseline value. Between-group comparisons will be tested using Wilcoxon rank sum tests. We will examine the distributions of the outcomes to determine whether parametric tests can be used in a definitive phase III trial. We will conduct all primary analyses according to the intention to treat paradigm. Exploratory analyses will include fitting ordinary least squares regression models to evaluate the association between the primary outcomes and a variety of possible predictors (e.g., baseline T-scores, use of calcium and vitamin D supplements, activity level, Tai Chi compliance, exposure to specific types of Tai Chi exercises, etc.). The goals are to identify factors that need to be included in the design of a definitive study (i.e., stratification factors) and to identify interesting associations for further investigation (i.e., hypothesis generation).

Specific Aim 3. To collect preliminary data evaluating the biomechanical and physiological mechanisms through which Tai Chi may impact bone turnover and fracture risks associated with osteopenia. Changes between baseline and 3 and 9 months in biomechanical parameters associated balance control during quiet standing, and joint kinetics and dynamic balance during chair rise and gait will be compared using signed rank tests. Similar analyses will be employed for clinical balance and function tests, as well as HRQOL and fear of falling. We will calculate Spearman correlations between measures of biomechanical parameters and measures of BMD changes (CTX, OSC, BMD) to evaluate the relationships between these domains. Based on these analyses, we will conduct exploratory regression analyses to explore mechanistic pathways to generate hypotheses for future validation.

#### Sample size and statistical power

There were no studies of osteopenic women that employed a 9 month Tai Chi intervention that we could use to estimate sample size and statistical power for the outcomes of interest. The closest approximation we found was the study by Yamazaki [53] which: a) specifically targeted osteopenic women; b) included short-term measures of bone turnover rates (CTX at 3, 6, and 12 months) and BMD (6 and 12 month); and c) was a based on a relatively low impact weight bearing exercise like Tai Chi (i.e. walking). In this study, Yamazaki observed a mean reduction in CTX at 6 months of 21% with a standard deviation of approximately 35% in the exercise group and a mean change of 0% with a standard deviation of approximately 20% in the control group. Assuming the same standard deviations, a sample size of 34 per group would be required to provide power of 0.80 to detect a 20% difference between groups (e.g., 0% change in control vs. 20% change for Tai Chi), with a parametric test. For at least some of the outcomes we expect data to be skewed. Therefore, to be conservative we will use non-parametric tests, which will result in a loss of efficiency that we estimate to be 5%. Allowing for 15% of participants to fail to complete the 9-month evaluation (based on our prior experience), we require 43 participants per group to ensure adequate power.

Yamazaki also measured percent change in lumbar BMD from baseline to 6 and 12 months [53]. At six months they observed changes of 0.47% ± 1.09% in the exercise group and -0.45% ± 1.20% in the control group. At 12 months, these changes were 1.71% ± 4.42% in the exercise group and -1.92% ± 2.94% in the control group. Using the same assumptions as above, our sample will provide power of 0.60 to detect a difference between groups of 0.90% at 9 months.

## Discussion

Results of this study will provide preliminary evidence regarding the value of Tai Chi as an intervention for decreasing fracture risk in osteopenic women. They will also inform the feasibility, value and potential limitations related to the use of pragmatic designs for the study of Tai Chi and related mind-body exercise. If the results are positive, this will help focus future, more in-depth, research on the most promising potential mechanisms of action identified by this study.

## Competing interests

DPK has received grants from Amgen, Merck, Novartis, Wyeth, and Hologic; served as a consultant for Amgen, Merck, Novartis, GSK, Philips Lifeline, Wyeth, P&G, and Lilly; and been a speaker for Novartis, Merck, and GSK. All other authors declare that they have no competing interests.

## Authors' contributions

PMW, JEB, RBD, and DPK participated in the conception of the trial and in plans for the analysis of the data. PB and BP had oversight for the biomotion substudy, CJC and MF facilitated patient recruitment, and EMC was responsible for data collection. PMW, DEK, and GYY, drafted the manuscript. All authors read and approved the final manuscript.

## Pre-publication history

The pre-publication history for this paper can be accessed here:

http://www.biomedcentral.com/1471-2474/11/40/prepub
